# Bacteriological and mechanical impact of the Sterrad sterilization method on personalized 3D printed guides for mandibular reconstruction

**DOI:** 10.1038/s41598-020-79752-7

**Published:** 2021-01-12

**Authors:** Romain Bosc, Lionel Tortolano, Barbara Hersant, Moussa Oudjhani, Céline Leplay, Paul L. Woerther, Paola Aguilar, Ronan Leguen, Jean-Paul Meningaud

**Affiliations:** 1 Department of Plastic, Reconstructive, Aesthetic and Maxillofacial Surgery, Henri Mondor Hospital, 51 avenue du Maréchal de Lattre de Tassigny, 94010 Créteil, France; 2 Henri Mondor Breast Center, Créteil, France; 3 Department of Pharmacy, Henri Mondor Hospital, Créteil, France; 4 EA 401 Matériaux et santé, Université Paris-Saclay, UFR Pharmacie, 92290 Châtenay Malabry, France; 5 Department of Microbiology and Infection Control, Henri Mondor Hospital, Créteil, France

**Keywords:** 3-D reconstruction, Implants, Reconstruction

## Abstract

Surgical cutting guides are increasingly used for maxillofacial reconstruction. They are usually provided by laboratories. In recent years, surgical teams have published studies on the possibility of manufacturing their own cutting guides thanks to 3D printers. The object of this study is to analyze the impact of the sterilization on the surface of those personalized models and to assess the effectiveness of sterilization. Using the data from high-resolution CT scan of patient, 3D models were generated through computerized assisted design and fabricated with a 3D printer using Acrylonitrile Butadiene Styrene (ABS). For the sterilization, a Sterrad method was used. In order to evaluate the effectiveness of sterilization, 3D models were artificially contaminated with several bacterial reference strains, sterilized and finally cultured. The surfaces and mechanical modifications were analyzed before and after sterilization with infrared spectrometry, surface contact angle, extensometer, scanning electron microscopy and atomic force microscopy. Ten models of different shapes and 24 samples were fabricated, sterilized and analyzed. The 3D models were designed in 48 h, printed in an average of 122 min and underwent a 47 min cycle of sterilization. All experimentally contaminated 3D models were negative in culture, with at least, a six log reduction of the initial inoculum. The hydrophobicity and roughness of the surface suffered few changes. The reproducibility of this procedure was proved by identical results in the three sterilization rounds. Using Sterrad process for the sterilization of ABS printed material doesn’t represent a bacterial risk for the patient. It is a feasible and safe innovative reconstructive method that can save time particularly for oncological cases.

## Introduction

Three-dimensional printing technology, also called additive manufacturing is having an increasing role in the medical field particularly for surgical planning and production of anatomical models, surgical instruments, implants and prostheses^1–3^.

In maxillofacial and reconstructive surgery, the challenge of is to restore the anatomical structure with the best aesthetic and functional aspect. Personalized surgery is essential in this territory^4^. 3D guides are the best tools to achieve an accurate, symmetrical and functional reconstruction.

The commercialization of low-cost 3D printers allowed us to internalize the 3D printing process and integrate it into our therapeutic plan avoiding a long and expensive external providing process^5^. Working with 3D printers implies the employment of several technologies: fused deposition modeling, stereolithography, selective laser sintering, and digital light processing. A diverse range of materials that are consistently evolving can be use in this printing process. In order to be positioned in open surgical wounds directly applied at the surface of the bone structure that will be cut or reshaped, these materials must have undergone an effective sterilization process. It is also essential to have a good assessment of the specific bacteriological risk associated with the use of these printed surgical guides. The ANSM (Agence Nationale de Sécurité du Médicament), which is the French equivalent of the FDA (Food and Drug Administration) has established a classification for medical devices with specific guidelines for sterilization procedures corresponding to their proximity to sterile tissue. The 3D printed guides fall into the class IIa (Class I for the FDA) of medical devices and therefore require fulfilling a specific set of security criteria in terms of sterilization^6^.

In the present study, the surgical guides were printed by the fused deposition technique with pure Acrylonitrile Butadiene Styrene (ABS). ABS has a glass transition temperature close to 105 °C (121°F) that is incompatible with classic steam autoclave sterilization (121 °C (250**°**F) for 18 min). The alternative could be a chemical sterilization process at low temperature with hydrogen peroxide: the Sterrad^7^. A study by Peniston et al*.*^8^ showed that hydrogen peroxide plasma and ethylene acid can be used to sterilize polylactic acid (glass transition temperature: 55 °C, 131°F) without causing any significant change to their biomechanical properties. Diab-Elschahawi et al., emphasized the importance of cleaning medical devices before being exposed to a subsequent hydrogen peroxide sterilization process^9^. Although hydrogen peroxide plasma can induce some changing on polymer surfaces, no published studies assessing its impact on biomechanics and its bactericidal efficiency are available for ABS^10,11^.

The aim of this study was to assess if 3D printed material used as medical device temporarily applied in surgical wounds can be effectively sterilized with the Sterrad method without alteration of the final shape, surface and functionality.

## Material and methods

### Conception and printing

**Figure 1:**
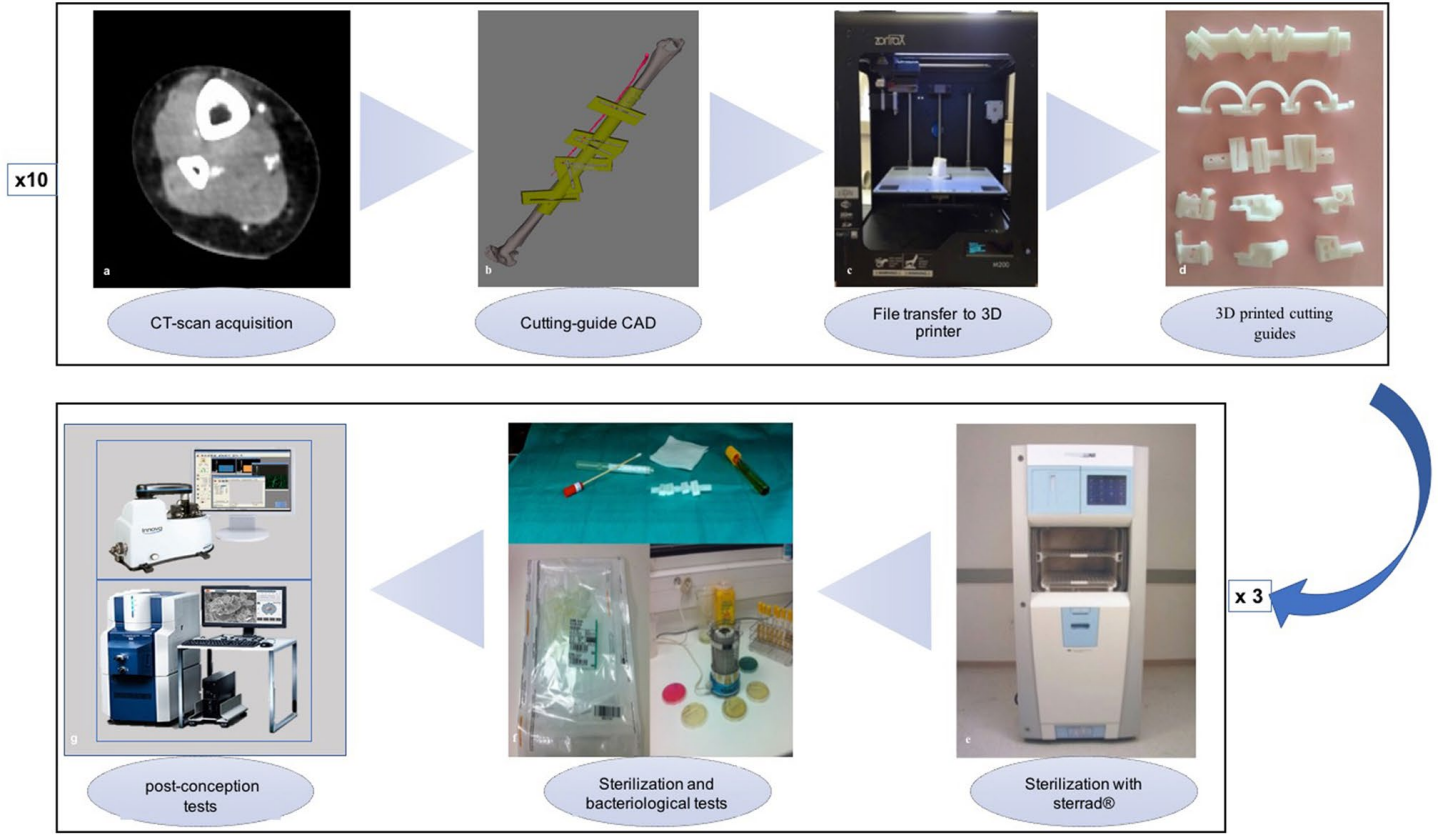
Manufacturing process of the surgical cutting guides with the ABS 3D printer. (**a**) DICOM format images from a CT-scan of the inferior limbs used for the volume rendering and 3D reconstruction, (**b**) Personalized 3D printed guides designed to anatomically fit the fibula and guide the osteotomies in order to have exactly the osseous segments needed for the mandibular reconstruction, and avoiding all important structures as vessels and nerves, (**c**) 3D printer to obtain ABS cutting guides from STL format digital files, (**d**) Cutting guides before sterilization, (**e**) The Sterrad 100NX is a system that allows dry, low temperature sterilization of heat-sensitive instruments using hydrogen peroxide vapor gas plasma technology without leaving any toxic residues. (**f**) Bacteriological tests after sterilization, (**g**) Surface analysis of the 3D printed samples.

Figure 1 summarizes the experimental protocol carried out by a trained plastic surgeon. For the conception of the digital model of the guides, we have used, for each case, anonymized high-resolution CT data in DICOM format (Fig. 1a) that was transformed into Standard Tesselation Language (STL) format and transferred to a computer 3D modeling software (MeshMixer 2: Autodesk, Inc) for the image segmentation and the generation of the digital 3D models^12–14^ (Fig. 1b).

The 3D models were sliced with the printer software Z-Suite with the following settings: ABS material, 0.14 mm of layer thickness, normal speed, full infill, support lite and autoprint cooling (Fig. 1c).

The 3D printer used was the Zortrax M200 that converted the digital model into a physical object by layer-to-layer deposition method using ABS (Fig. 1d). This 3D printing technique consists in liquefying the ABS by heating it beyond its melting point, and then pushing it through an extruder mounted on a movable gantry that moves according to the instructions generated by the slicing program. By laying down, layer after layer that rapidly cools and bond to each other, the digital model is thus converted into a physical model (Fig. 1d). The time needed for each step of the conception of the cut guides was also recorded.

### Sterilization

After the fabrication, and once the support was taken out manually, the guides were treated with a chemical sterilization process at low temperature with hydrogen peroxide: the Sterrad 100NX (ASP c/o Ethicon SAS, Issy les Moulineaux, Fr) (Fig. 1e). All guides underwent before going into the Sterrad a classic 15 min manual wash with a detergent made of didecyldimethylammonium chlorure, digluconate chlorhexidine and non-ionic surfactants (Anios’clean EXCEL D at 5 mL/1 L of water, Anios, France), a 5 min sonication, drying at room temperature with clean medical air and packaging into a double specific Sterrad bag. The Sterrad 100NX is based on 58–59.5% aqueous hydrogen peroxide, which is concentrated to about 95% through removal of water from the peroxide solution before evaporation. The standard cycle sterilizer-setting intended for the sterilization of most surgical instruments was chosen for our experiments. We use the standard Sterrad cycle of 47 min that is the cycle used for most heat-sensitive surgical instruments.

In order to validate each batch and according to ISO recommendations, a microbiological control: Attest (3 M, Cergy-Pontoise); containing spores of *Geobacillus stearothermophilus* undergoes the sterilization cycle with the Sterrad method (Fig. 1f)^15^.

### Surface analysis (Fig. ***1******g)***

Textured samples have been printed for surface analysis. Samples were analyzed before and after sterilization.

#### Contact angle

Contact angle measurements were carried out using a Digidrop apparatus from GBX (Bourg de Péage, France) by doing static contact angle measurements with a water drop.

#### Fourier-transform infrared spectroscopy (FT-IR)

The spectrometer apparatus was a Perkin-Elmer (Courtaboeuf, France) Spectrum 2000. It was used in transmission mode directly on the extruded film (disposed perpendicularly to the beam). The wavelength range was set from 4000 to 400 cm^−1^ with a resolution of 4 cm^−1^ during 16 scans. The styrene (1490 cm^−1^)/carbonyl (1733 cm^−1^) and the one between carbonyl and polybutadiene at 966 cm^−1^ were studied.

#### Scanning electron microscope

Surface observations was carried out using a Hitachi Flex Sem 100 operating at 15 kV equipped with secondary electron detector (SE) with a magnification factor × 55, × 250 and × 1000.

#### Atomic force microscopy

Surface roughness of the implants was analyzed with atomic force microscopy (AFM). Measurements were performed in ambient air with an Innova AFM (Bruker, France) using the tapping mode. The cantilever resonant frequency was 320 kHz using a silicon probe tip with a nominal spring constant of 42 N/m (NCHV-A, Bruker) and a radius of 10 nm. The scan rate was 0.5 Hz. The root mean squared roughness (Rq), as well as the profile were calculated using the Gwyddion 2.35 software.

### Mechanical properties

The mechanical properties of ABS before and after sterilization were measured with an AGS-X extensometer (Shimadzu). The elongation kinetics was 50 mm/min. The force needs to reach the breaking point and the Young’s modulus were recorded with Trapezium X software. Two batch of ABS were tested. 5 samples before non-sterile and 5 samples sterile were used. All samples were considered as the worst cases material (rectangular band of 2 mm thickness, 15 mm width and 60 mm length).

### Sterilization effectiveness (Fig. ***2******)***

**Figure 2:**
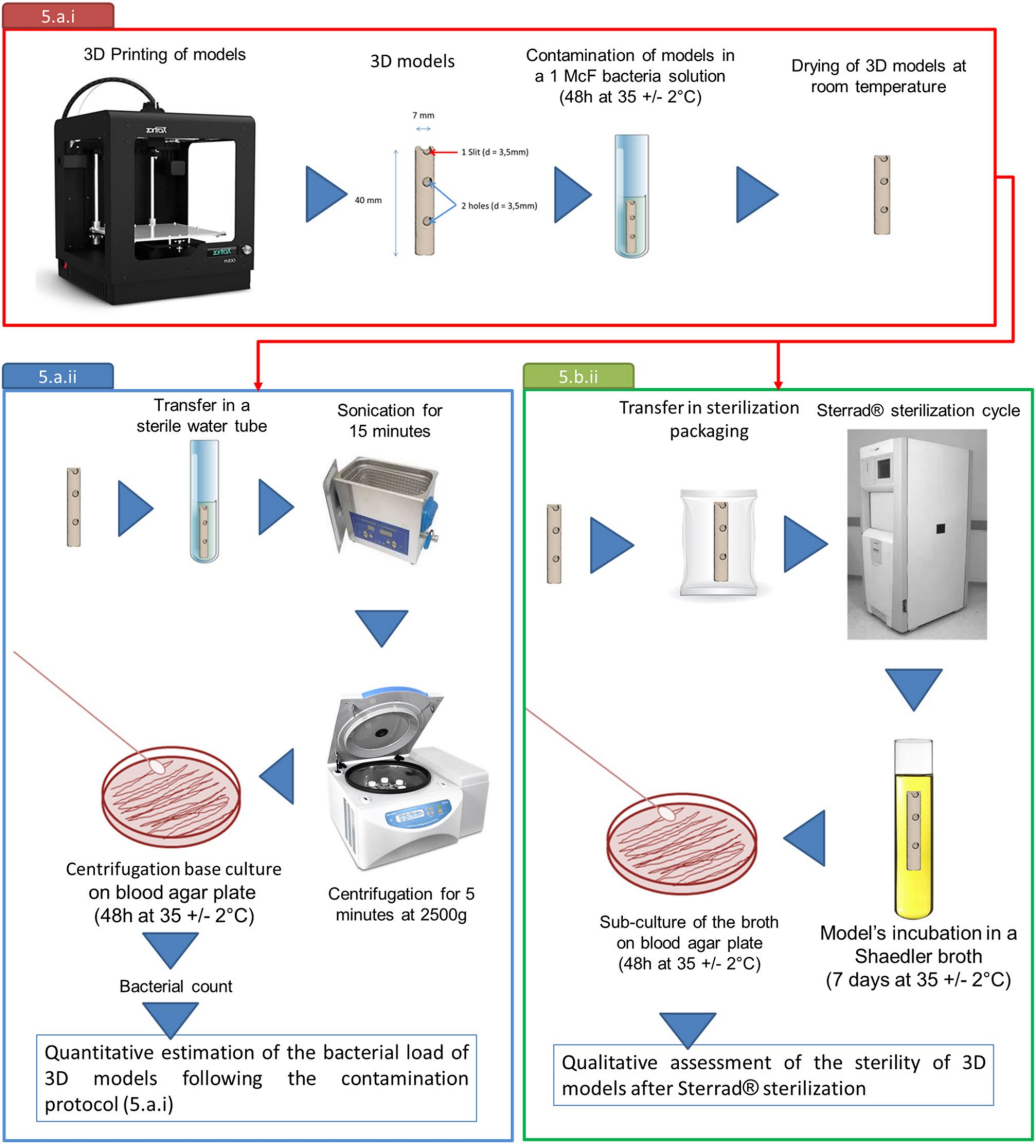
Steps of the in-house to assess the effectiveness of Sterrad's sterilization process.

In order to measure the effectiveness of the Sterrad method for sterilizing 3D models, specific models have been designed and artificially contaminated with several bacterial species. Afterwards, the bacterial load on the surface of these models and the effectiveness of the sterilization protocol were assessed in parallel. A preliminary study was carried out to determine the sensitivity of the Schadler broths used to assess the sterilization effectiveness.

#### Contamination of the 3D models

##### Contamination protocol

In order to assess the efficiency of sterilization, 3D specific models were artificially contaminated with four different reference bacterial strains: *E. coli* (ATCC 25922), *S. aureus* (ATCC 29213), *E. faecalis* (ATCC 29212) and *P. aeruginosa* (ATCC 27853). The models were immersed in a suspension calibrated at 1 McFarland (≈ 3.10^8^ bacteria/ml) during 48 h at 35 ± 2 °C. After incubation, the models were removed from the 1 McFarland solutions and dried at room temperature to undergo immediately the protocols 5.a.ii or 5.b.ii.

##### Estimation of the bacterial load on 3D models surface after contamination protocol

Twelve 3D models (tree for each strain) were artificially contaminated following the contamination protocol (5.a.i). After incubation, the models were removed from the 1 McFarland solutions, dried at room temperature and transferred into a sterile water tube. Then, the tubes were placed in an ultrasonic tank (Bandelin, Berlin, Germany) for 15 min (frequency 35 kHz, power 160 W) in order to remove the bacteria from the 3D models^16^. After centrifugation (2500 *g*, 5 min) and removal of the supernatant, the centrifugation base was dissolved in 1 ml and 10 µl were isolated on blood agar (Biorad, Marne-la-coquette, France). After 48 h incubation at 35 ± 2 °C, the colonies were counted.

#### Assessment of Sterrad sterilization protocol

##### Preliminary evaluation of the sensitivity of Schaedler broths (supplementary data)

For each strain, 100 µl of bacterial suspensions, approximately calibrated to 10^3^ 10^2^ and 10 CFU/ml were introduced in triplicate in a Schaedler broth. The actual concentrations were simultaneously verified on blood agar culture in triplicate (Supplementary data). The broths were incubated at 35 ± 2 °C and checked at, 24 h, 48 h, 5 and 7 days. After 48 h of culture, sub-cultures with 100 µl of each broth were performed on blood agar plates and incubated at 48 h at 35 ± 2 °C. A second subculture, performed under the same conditions, was carried out after 7 days of broth incubation, when the first one was negative.

##### Assessment of sterilization effectiveness

Twenty-four 3D models (6 for each strain) were artificially contaminated following the contamination protocol (5.a.i). In each case, one of the six models was used as growth control and was directly incubated in Schaedler broth (Biomerieux, Marcy-l'Étoile, France) at 35 °C + /− 2 °C for 48 h, whereas the five other models underwent the different sterilization steps (see above). After sterilization, each model was incubated in a Schaedler broth at 35 °C + /− 2 °C until clouding or for a total of seven days. Afterwards, 100 µl of each model broth (growth control and sterilized models) were sub-cultured on blood agar plates and have been read after 48 h incubation at 35 + /− 2 °C. This in-house protocol was carried out in triplicate.

### Statistic analysis

Categorical variables are presented as counts and percentages and were compared by means of the Fisher’s exact test. Continuous variables following a normal distribution are presented as mean ± Standard Deviation (SD) and were compared with a Student t test with 8 degrees of freedom (dof). A two-way variance analysis (ANOVA) test was applied to compare the roughness differences between all three samples before and after sterilization. All p-values are two-sided and a value of *p* < 0.05 was considered significant. All analyses were performed with the use of PRISM, version 7 (Graph Pad, USA). All the authors had full access to all of the data and take full responsibility for the integrity of the data and the accuracy of the data analysis. All methods were carried out in accordance with relevant guidelines and regulations without experiment on humans or animals. No member of our research team named in the author list of the paper had access to identifying patient’s information when analyzing the data.

## Results

The same surgeon from the Plastic Surgery department completed the generation and design, then the 3D printing of the ten models. The 3D printed models used were 10 cutting guides for fibular and mandibular osteotomies used for oral cancer and mandibular reconstruction with a free fibular flap. These guides had different complex shapes including holes, narrow corners, and thin arches. The size of both holes and slots were adapted to the size of the screws (3.5 × 12 mm) and the thickness of the oscillating saws (0.3 mm) used in usual clinical practice.

The 3D models were printed in an average time of 122 min. We did not encounter any technical problem through the fabrication process. The sterilization program lasted for each round 47 min.

### Surface contact angle

Before the sterilization the surface contact angle measure with water at room temperature was about 70.2 °C. After sterilization, the SCA increase to 82.3 °C revealing highest surface tension as before treatment by Sterrad. This difference was statistically significant (t test of student, α = 0.05; *p* < 0.001).

**Figure 3:**
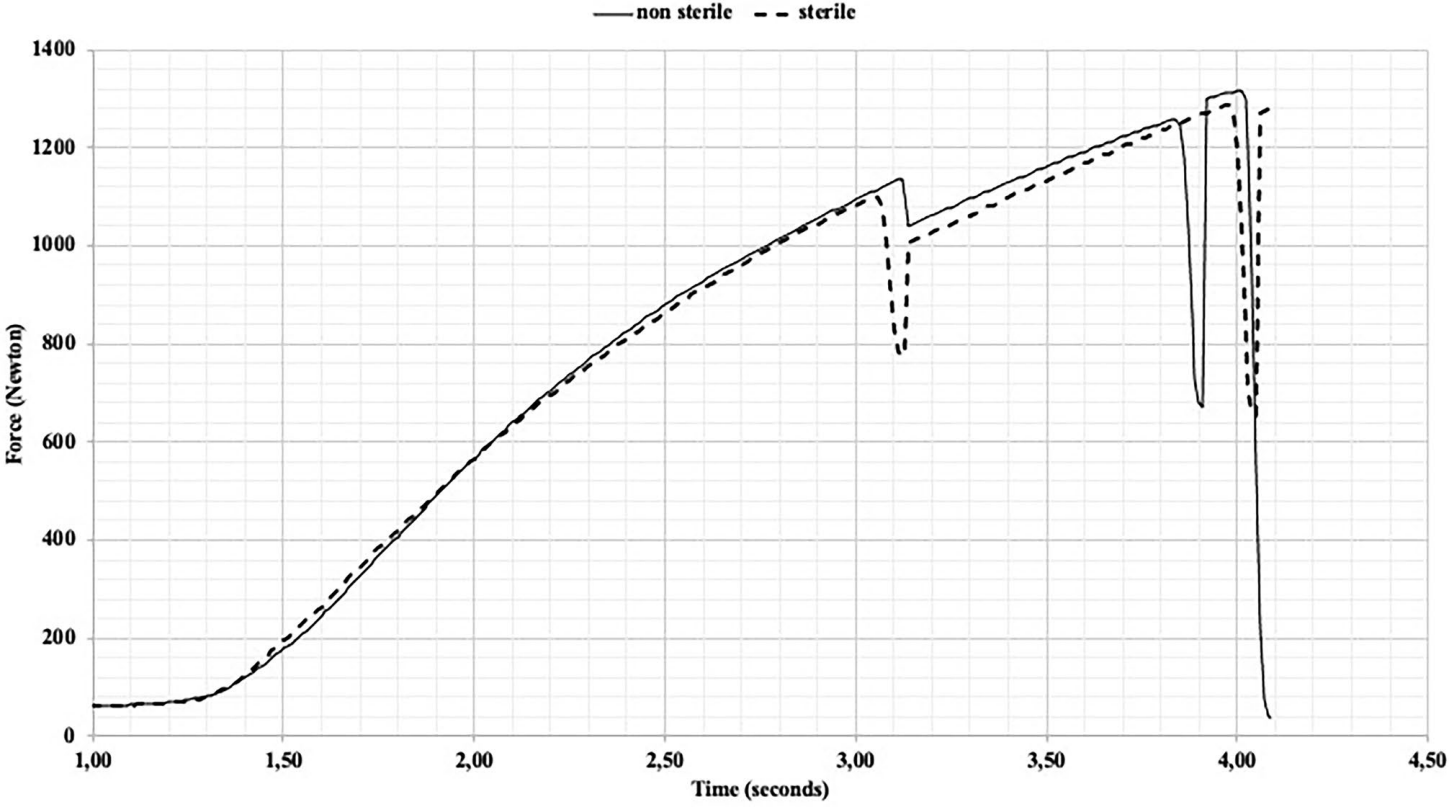
Mean profiles of the mechanical properties of the models tested.

The Fig. 3 shows the mean profiles of the mechanical properties of the models tested. The Young’s modulus was equivalent in all samples from the batch production with or without sterilization. A non-significant difference of the strength needed to reach the breaking point was calculated. Breaking strengths were 1304 N for non-sterile bands of ABS and 1388 N for sterilized bands (t test of student 8 dof, α 5%, *p* 0.12). Also, a non-significant difference was calculated for the elongation before. Elongations were 3.08 mm for non-sterile ABS band and 3.23 mm for sterilized bands (t test of student 8 dof, α 5%, *p* 0.53).

**Figure 4:**
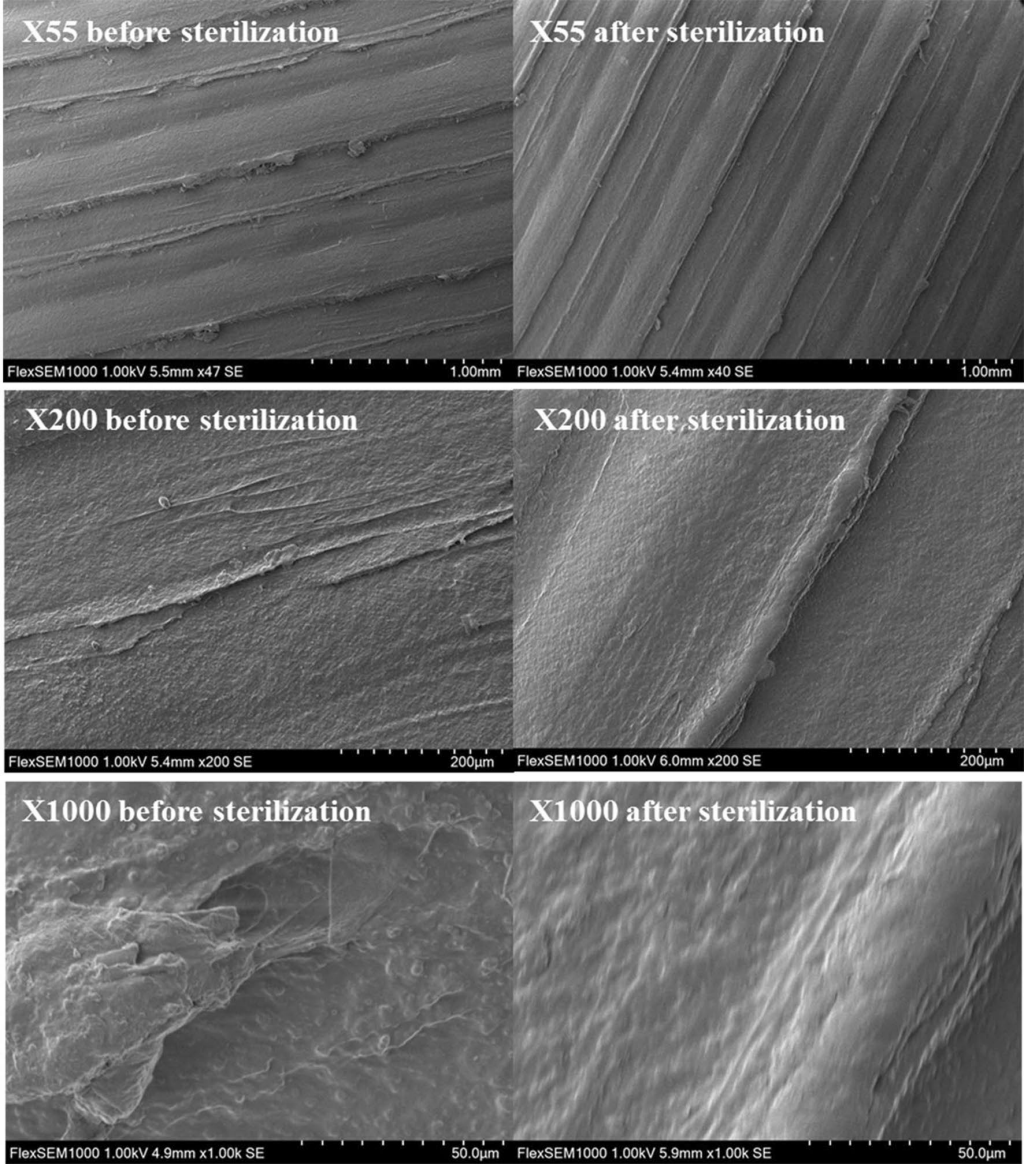
After sterilization (right side), surface observe with SEM were smother as before, with some few topography changes.

The sterilization of the ABS did not induce strong changes of the surface. For the FT-IR spectrum, the ratios of each signal were the same. The styrene/carbonyl ratio was not significantly different between samples before and after sterilization (t test of student, α = 0.05; *p* = 0.127). The butadiene/carbonyl ratio did not significantly change after sterilization (t test of student, α = 0.05; *p* = 0.764).

Profile modifications were analyzed by SEM from × 55 magnification to × 1000.

The sterilization did not change the appearance of the profiles of the surfaces made of regular ABS-polymer lines. Nevertheless, some gaps in the lines of impression were observed at × 200 magnification. This was identified as the consequences of artifacts during the molding process. These artifacts were about 100 to 300 µm. At a highest magnification (× 1000), the roughness of the impression appeared. Although these textures were under 50 µm, their number decreased after sterilization.

### AFM

All samples had homogeneous surface properties as for the mean square roughness (Rq) or the surface profiles. Texture was made of regular peaks of approximately 2 µm high and 20–25 µm width.

There was no roughness difference between all three samples before and after sterilization (A two-way ANOVA, α = 0.05) nevertheless profiles were less regular as before the sterilization. These observations confirmed those made with the SEM.

### Sterilization effectiveness

#### Preliminary evaluation of the sensitivity of Schaedler broths

For the lowest concentration of *E. coli* (ATCC 25922), *S. aureus* (ATCC 29213), *E. faecalis* (ATCC 29212) and *P. aeruginosa* (ATCC 27853) suspension, the actual concentrations were respectively 6.6, 3.33, 20 and 13 CFU/ml. The theoretical number of bacteria present in 100 µl inoculated into the broths was respectively 0.66, 0.33, 2 and 1.3. After 7 days of incubation, 2/3, 1/3, 3/3 and 2/3 of the subcultures corresponding respectively to the 4 tested strains were positive (Supplementary data).

#### Estimation of the bacterial load on 3D models surface

The bacterial load on the surface of the models after 48 h of incubation was estimated at 6.6*10^4^ for *E. coli* models, 3.3*10^6^ for *S. aureus* models and 10^7^ for *E. faecalis* and *P. aeruginosa* models (Table 1).

**Table 1:** Estimation of the bacterial load on the surface of the 3D models carried out directly after the 48-h-contamination protocol with four bacterial reference strain.

Contaminating reference strain	Colony count on Subculture 1	Colony count on Subculture 2	Colony count on Subculture 3	Average number of bacteria removed of the 3D models
*E. coli* ATCC 25922 (n = 3)	10^5^	10^4^	10^5^	6.6*10^4^
*S. aureus* ATCC 29213 (n = 3)	10^6^	10^7^	10^6^	3.3*10^6^
*E. faecalis* ATCC 29212 (n = 3)	10^7^	10^7^	10^7^	10^7^
*P. aeruginosa* ATCC 27853 (n = 3)	10^7^	10^7^	10^7^	10^7^

#### Assessment of sterilization effectiveness

All internal controls of the Sterrad sterilization cycle, including Attest control, were successfully completed. All subcultures of the growth control broths were positive. None of the Schaedler broths containing the sterilized 3D models were cloudy after 7 days of incubation. Moreover, all the corresponding subcultures incubated for 48 h were also negative (Table 2).

**Table 2:** Triplicate Schaedler broth cultures after Sterrad sterilization of contaminated 3D models with the 48-h-contamination protocol.

Contaminating reference strain	Growth control Subculture on agar at 48 h	Schaedler broths Turbidity at 7 days	7-days-Schaedler broths subcultures on blood agar after 48 h
E. coli ATCC 25922 (n = 6 × 3)	Positive (3/3)	Clear (15/15)	Negative (15/15)
S. aureus ATCC 29213 (n = 6 × 3)	Positive (3/3)	Clear (15/15)	Negative (15/15)
*E. faecalis* ATCC 29212 (n = 6 × 3)	Positive (3/3)	Clear (15/15)	Negative (15/15)
*P. aeruginosa* ATCC 27853 (n = 6 × 3)	Positive (3/3)	Clear (15/15)	Negative (15/15)

Furthermore, after sterilization the 3D guides didn’t show any macroscopic alteration in terms of shape, structure or hardness.

## Discussion

This preliminary study shows that the Sterrad process enables to obtain complete sterilization of the cutting guides manufactured in ABS without altering their morphology, with acceptable surface modifications.

Personalized surgery is essential to practice, more so in the maxillofacial field where reconstruction has to be as patient specific as possible to gain in symmetry and functionality, allowing a better quality of life.

Authors believe that virtual planning technologies are an emerging criterion standard in mandible reconstruction^13,14,17^. The use of computer assisted designed osteotomy guides for helping mandible and maxilla reconstruction has been demonstrated since more than 20 years^17,18^. A study on “in house” manufacturing of bone cut guides has been already published with an evaluation of clinical outcomes^12^. The results showed that the rates of complications and post-operative infections were not different than before the use of 3D printing. Over the past ten years, many authors have published mandibular reconstruction cases with the help of a model based on rapid prototyping technology, which assists accurate contouring of plates and/or planning of bone graft harvest geometry before surgery^19^.

The 3D personalized guided surgery is not performed in many hospitals because of the hefty fee of the 3D guides that commercial companies providing those services set up and the delay of average three weeks to have the guides via those companies. The software programs that we used are all free of charge and downloadable via the internet. Surgeons of our maxillofacial department made the conception and production of the guides without specialized training in either computer 3D modeling or engineering. The downside of the in-doors guide production in that it is time consuming^5^ so we could imagine that it should be ideally be place into the hands of a specific hospital technician.

Numerous materials are available for fused deposition printing. We tested several materials: with glass and PLA (polylactid acid) we encountered issues as obstruction of the extruder, and fragility of the guides, with ABS we didn’t had any technical problem in the printing process. Moreover, post-processing tasks after printing are very simple and can be performed directly in the sterilization room without complex equipment.

The guides are used directly into the body of the patient, applying them to the bone, therefore they are considered as a “critical item” meaning they are associated with a high risk of infection if they are contaminated with any microorganism. It is recommended for critical items^8^ that are heat sensitive to be sterilized by chemical process as hydrogen peroxide gas plasma.

In our hospital two types of sterilization process are used: the steam autoclave and the Sterrad. There is no standard procedure for the sterilization of this ABS medical device.

We chose the Sterrad system because it combines the use of hydrogen peroxide vapor and plasma safely to rapidly sterilize most medical heat-labile instruments and it is known to be less prone to affect fragile material as endoscopic tools.

This study shows that our process of printing, hand washing, and hydrogen peroxide gas plasma sterilization is effective in terms of bacteriological risk without macroscopic shape changes and few surface structure modifications^20^. The surface analyses show a regular high roughness and hydrophobicity that could both explain the initial bacterial colonization before sterilization. The sterilization procedure has increased the hydrophobicity but has decreased the global roughness in the same time. The first observation is reliable to the oxidation of the superficial layers of the ABS material that could have changed the surface energy on the material and, thereby its interaction with water drop^21^. A higher hydrophobicity is a risk of bacterial colonization. The roughness changes could be attributable to the friction of the handwashing on polymer surface. Nevertheless, smoother surfaces have less bacterial colonization than rough surfaces. Moreover, in term on biocompatibility, smoother surface present less risk than rough ones^22,23^.

The sterilization by plasma gas has not a significant impact on mechanical properties tested of ABS polymer. We estime that the non-significant modifications of both elongation and breaking point (Fig. 3a,b) are tolerable because they could increase the strength of the device for the surgery. Moreover, the difference observed seems to be more dependent of the batch of production than the sterilization of the polymer.

To study the effectiveness of the Sterrad method on bacterial colonisation of our models, we created an in-house experimental protocol with four reference strains from clinically relevant species^24^. According to Standard 11737-2:2019^25^, the recommended method to control the sterilization process is the immersion of the sterilized product in a liquid culture medium. However, this standard does not specify which type of medium should be used. Schaedler broth have been chosen for its ability to grow fastidious bacteria, including anaerobes, and its use in the diagnosis of prosthetic infections^26,27^. Moreover, the Schaedler broth also allows the culture of *E. coli, P. aeruginosa, E. faecalis* and *S. aureus* species, according to previous publications^28–30^. The preliminary study aiming to assess the sensitivity of Schaedler broths shows a good match between the theoretical number of bacteria inoculated and the proportion of positive schaedler broth with the lowest bacterial incolum. Whichever the tested strain, the detection threshold for Schaedler broth was close to 1 bacteria, attesting the good sensitivity of our protocol. It also demonstrated its capacity to grow the strains used in this study.

The differences observed between the models contaminated with *E. coli* and those contaminated with the other strains may reflect discrepancies between the contamination abilities of the tested strains. Indeed, we can hypothesize that the *E. coli* strain was less able to colonize the 3D models and thus be present in lesser concentration.

Quantification of the bacterial load on the surface of the 3D models, in parallel with the negativity of the cultures and the determination of the threshold of the Schaedler broth makes it possible to estimate that sterilization by the Sterrad method gives, at least, a 6 log reduction of the initial inoculum for the *S. aureus, E. faecalis* and *P. aeruginosa* contamination protocol. Concerning the *E. coli* contamination protocol, an approximate 5 log reduction of the bacterial load was observed, due to a lower initial inoculum. Altogether, the Sterrad sterilization method appears to provide good conditions for material implantation, even in case of very high bacterial inoculum.

This study has several limitations. First, the mechanical tests used to investigate the properties of the 3D models before and after the sterilization step were focused on the research of modifications of both elasticity and breaking points of the 3D models printed in ABS. But there are many others mechanical properties which could be tested, and which could give information about the hardness of the final product. Since the guides are used to guide the saw during osteotomies, we expect them to have high strength and that they retain it after sterilization. But the clinical studies already published with this material did not show any fragility of the guides after sterilization, when they were used in the operating room. This is one of the important issues for clinical transfer that needs to be assessed in future studies.

Second, only one shape of model was used to assess the sterilization efficiency. This model included a slit and two holes to simulate the complexity of the 3D models used in practice, while being small enough to be immersed in a tube of Schaedler broth. However, other shapes, with a higher level of reality should be tested to confirm our results.

Third, the statistical power to determine the detection threshold of the Schaedler broth is low; a test on a larger sample size would allow a more accurate estimate of this parameter.

Fourth, the ability of sonication to detach all bacteria from the 3D models is uncertain, leading to a possible underestimation of the initial inoculum*.*

Fifth, as defined in the ISO 11138-7^15^ standard, the sterilization assurance level (SAL) corresponds to the probability that a single unit will not be sterile at the end of a sterilization cycle. The commonly SAL accepted is 10^–6^. Given the complete reduction of the starting inoculum, the calculation of this indicator was impossible with this protocol. A concentration gradient greater than 1McFarland should be tested to calculate the SAL. However, the reduction of inoculum was comparable to that of the internal control ATTEST, which contained at least 10^6^ spores of *G. stearothermophilus.*

Sixth, surface analysis was performed on 10 different cutting guides. Since the guides are personalized, they are different for each patient and for each surgical procedure. So, they have different shapes and masses. In order to obtain an experimental protocol that should be closer to the actual conditions of use of this material, we considered that it was wiser to carry out three series of tests on each guide rather than repeating the tests on a single guide printed in ten copies. However, such variations are also likely to occur in the clinical practice.

In contrast, manufacturing conditions and print parameters of the guides were strictly the same: layer thickness, ABS density and print speed. 3D printing was always performed in the operating room in a dedicated room with controlled airflow.

Seventh, we performed this study with cutting guides designed by a single surgeon. It is likely that the design and the shapes of 3D models could vary with other operators due to differences in cutting guides usage. This is another important issue for clinical transfer that needs to be assessed in future studies.

## Conclusion

In this study, we have shown that it is feasible to fabricate with the hospital’s resources an anatomically accurate patient specific guide by using a low-cost 3D printer and a specific Sterrad sterilization program. Even if in-house three-dimensional printing with ABS is feasible, affordable and may represent a gain of time with an acceptable bacteriological added risk, we cannot extend this process to other kind of materials for 3D printers and we think that all new material should be specifically tested.

## Supplementary Information

Supplementary Information 1.

Supplementary Information 2.
