# Supplementary material for: Bacteriological and mechanical impact of the Sterrad sterilization method on personalized 3D printed guides for mandibular reconstruction
Source: Sci Rep. 2021 Jan 12;11:581. doi: 10.1038/s41598-020-79752-7 (PMC7804113; doi:10.1038/s41598-020-79752-7)
Supplement: Supplementary file 1 — Supplementary Information 1. [file 41598_2020_79752_MOESM1_ESM.docx]

**Title:** Bacteriological and mechanical impact of the Sterrad sterilization method on personalized 3D printed guides for mandibular reconstruction

# **Authors:** Romain Bosc^1,2^, MD, PHD; Lionel Tortolano^3,4^, PharmD, PHD; Barbara Hersant^1^, MD, PHD; Moussa Oudjhani^3^, PharmD; Céline Leplay^3^, PharmD; Paul L. Woerther^5^, MD; Paola Aguilar^1^, MD; Ronan Leguen^5^, MD; Jean-Paul Meningaud^1^, MD, PhD.

^1^Department of Plastic, Reconstructive, Aesthetic and maxillofacial Surgery, Henri Mondor Hospital, Creteil, France

^2^Henri Mondor Breast Center, Creteil, France

^3^Department of Pharmacy, Henri Mondor Hospital, Créteil, France

^4^EA 401 Matériaux et santé. Université Paris-Saclay, UFR Pharmacie Châtenay Malabry F 92290

^5^Department of Microbiology and Infection Control, Henri Mondor Hospital, Créteil, France

**Corresponding author contact information :**

Dr Romain Bosc, M.D., Ph.D.

ORCID ID : 0000-0003-3867-8135

51 avenue du Maréchal de Lattre de Tassigny, Créteil 94010, France

Email : romainbosc@gmail.com

Tel : 33 1 49 81 25 33

Fax : 33 1 49 81 25 32

**Acknowledgements:** The authors would like to thank the central pharmacy and the bacteriologic department of the Centre Hospitalier Universitaire Henri Mondor for their contribution.

Supplementary dataset 1

|  | Tested strains | | *E. coli* ATCC 25922 | | | *S. aureus* ATCC 29213 | | | *P. aeruginosa* ATCC 27853 | | | *E. faecalis* ATCC 29212 | | |
| --- | --- | --- | --- | --- | --- | --- | --- | --- | --- | --- | --- | --- | --- | --- |
| Calibration of the bacterial suspensions | Expected bacterial concentration of the supension | | 10.3 CFU/mL | 10.2 CFU/mL | 10 CFU/mL | 10.3 CFU/mL | 10.2 CFU/mL | 10 CFU/mL | 10.3 CFU/mL | 10.2 CFU/mL | 10 CFU/mL | 10.3 CFU/mL | 10.2 CFU/mL | 10 CFU/mL |
|  | Number of colony on the plate per 100µl of suspension | | 70 | 6 | 1 | 40 | 3 | 0 | 100 | 16 | 1 | 250 | 35 | 1 |
|  |  |  | 60 | 4 | 0 | 35 | 5 | 0 | 110 | 15 | 2 | 260 | 25 | 2 |
|  |  |  | 90 | 12 | 1 | 30 | 5 | 1 | 130 | 10 | 1 | 280 | 29 | 3 |
|  | Effective bacterial concentration | | 7,3*10.2 CFU/mL | 73,3 CFU/mL | 6,6 CFU/mL | 3,5*10.2 CFU/mL | 43,3 CFU/mL | 3,33 CFU/mL | 1,1*10.3 CFU/mL | 1,4*10.2 CFU/mL | 13 CFU/mL | 2,6*10.3 CFU/mL | 3*10.2 CFU/mL | 20 CFU/mL |
| Culture results of 100 µl of each suspension in a Schaedler Broth (triplicate) | Theoretical number of bacteria inoculated into the Schaedler broth (in 100 µl of each suspensions) | | 73 | 7,3 | 0,66 | 35 | 4,3 | 0,33 | 100 | 14 | 1,3 | 260 | 30 | 2 |
|  | Schaedler n°1 | Blurry time | 24h | 24h | 24h | 24h | 48h | no | 48h | 48h | 48h | 24h | 24h | 24h |
|  |  | 48h sub-culture | Positive | Positive | Positive | Positive | Positive | Negative | Positive | Positive | Positive | Positive | Positive | Positive |
|  |  | 7 days sub-cullture | Unrealized | Unrealized | Unrealized | Unrealized | Unrealized | Negative | Unrealized | Unrealized | Unrealized | Unrealized | Unrealized | Unrealized |
|  | Schaedler n°2 | Blurry time | 24h | 24h | 24h | 24h | 48h | no | 48h | 48h | 48h | 24h | 24h | 24h |
|  |  | 48h sub-culture | Positive | Positive | Positive | Positive | Positive | Negative | Positive | Positive | Positive | Positive | Positive | Positive |
|  |  | 7 days sub-cullture | Unrealized | Unrealized | Unrealized | Unrealized | Unrealized | Negative | Unrealized | Unrealized | Unrealized | Unrealized | Unrealized | Unrealized |
|  | Schaedler n°3 | Blurry time | 24h | 24h | no | 24h | 48h | no | 48h | 48h | no | 24h | 24h | 24h |
|  |  | 48h sub-culture | Positive | Positive | Negative | Positive | Positive | Negative | Positive | Positive | Negative | Positive | Positive | Positive |
|  |  | 7 days sub-cullture | Unrealized | Unrealized | Negative | Unrealized | Unrealized | Positive | Unrealized | Unrealized | Negative | Unrealized | Unrealized | Unrealized |

Table 1: Determination of the detection threshold of the Schaedler broth with the four bacterial reference strain.
