# Supplementary material for: Bacteriological and mechanical impact of the Sterrad sterilization method on personalized 3D printed guides for mandibular reconstruction
Source: Sci Rep. 2021 Jan 12;11:581. doi: 10.1038/s41598-020-79752-7 (PMC7804113; doi:10.1038/s41598-020-79752-7)
Supplement: Supplementary file 2 — Supplementary Information 2. [file 41598_2020_79752_MOESM2_ESM.docx]

**Title:** Bacteriological and mechanical impact of the Sterrad sterilization method on personalized 3D printed guides for mandibular reconstruction

# **Authors:** Romain Bosc^1,2^, MD, PHD; Lionel Tortolano^3,4^, PharmD, PHD; Barbara Hersant^1^, MD, PHD; Moussa Oudjhani^3^, PharmD; Céline Leplay^3^, PharmD; Paul L. Woerther^5^, MD; Paola Aguilar^1^, MD; Ronan Leguen^5^, MD; Jean-Paul Meningaud^1^, MD, PhD.

^1^Department of Plastic, Reconstructive, Aesthetic and maxillofacial Surgery, Henri Mondor Hospital, Creteil, France

^2^Henri Mondor Breast Center, Creteil, France

^3^Department of Pharmacy, Henri Mondor Hospital, Créteil, France

^4^EA 401 Matériaux et santé. Université Paris-Saclay, UFR Pharmacie Châtenay Malabry F 92290

^5^Department of Microbiology and Infection Control, Henri Mondor Hospital, Créteil, France

**Corresponding author contact information :**

Dr Romain Bosc, M.D., Ph.D.

ORCID ID : 0000-0003-3867-8135

51 avenue du Maréchal de Lattre de Tassigny, Créteil 94010, France

Email : romainbosc@gmail.com

Tel : 33 1 49 81 25 33

Fax : 33 1 49 81 25 32

**Acknowledgements:** The authors would like to thank the central pharmacy and the bacteriologic department of the Centre Hospitalier Universitaire Henri Mondor for their contribution.

Supplementary data :

Figure A


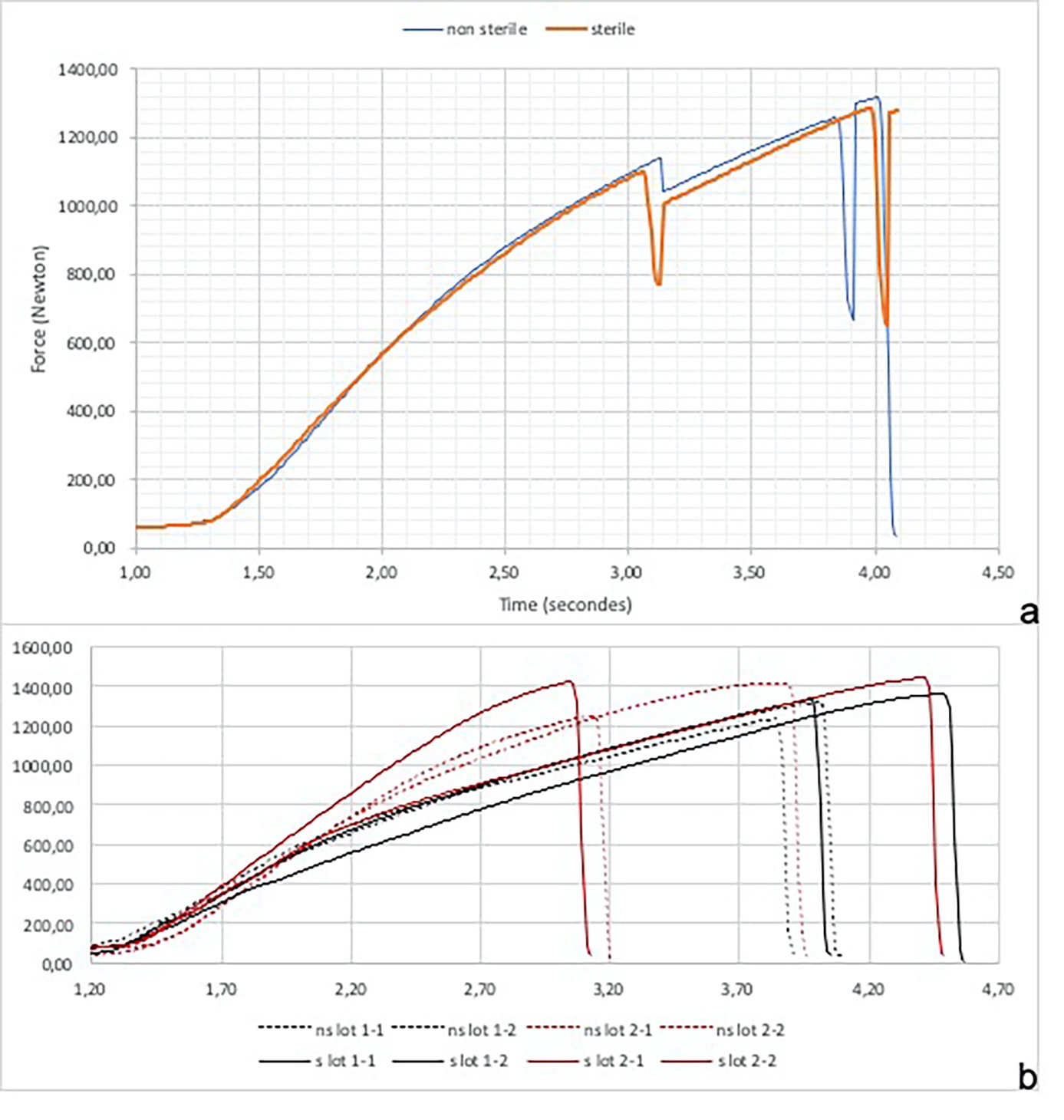


Figure A : Mean profiles (Aa) and details (Ab) of the mechanical properties of the models tested.


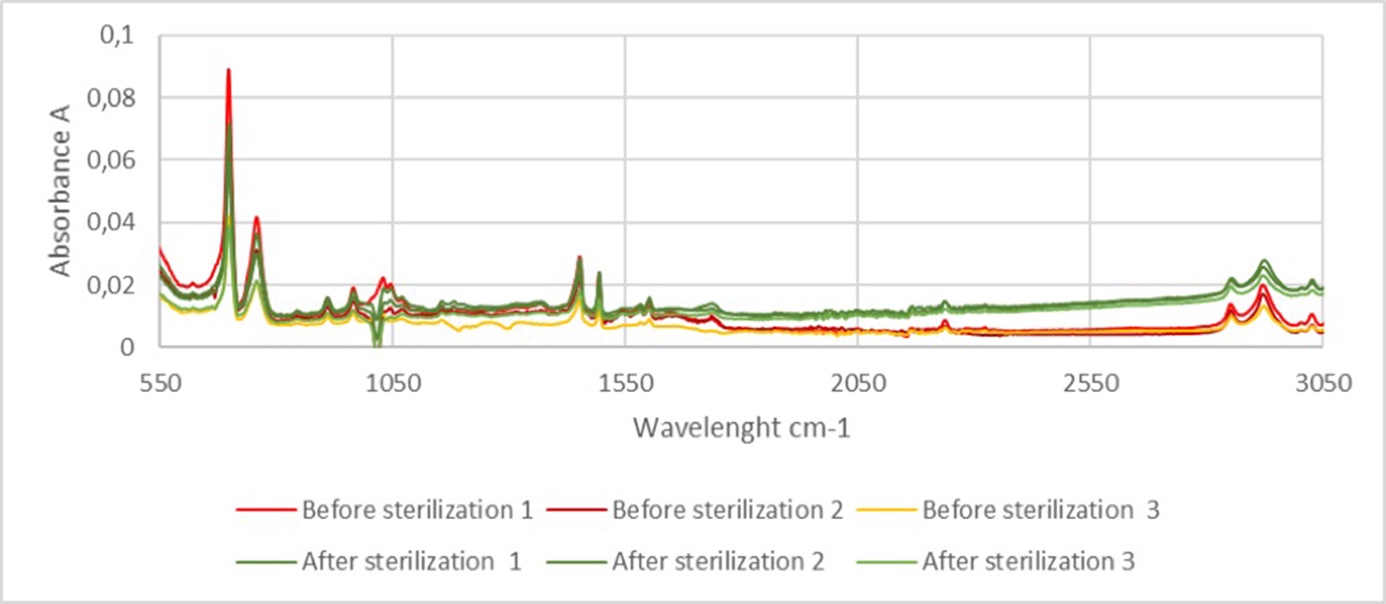
Figure B :Fourier-transform infrared spectroscopy (FT-IR): The styrene/carbonyl ratio is not significantly different between samples before and after sterilization.


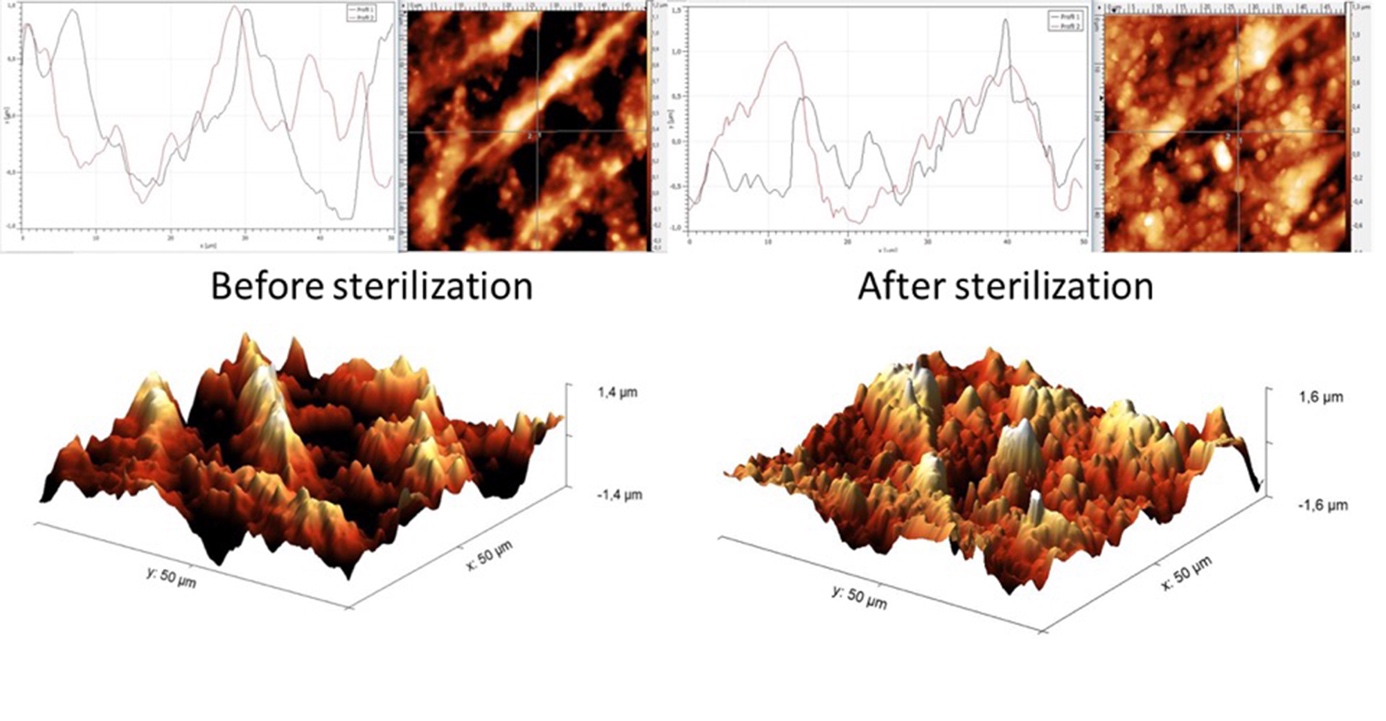
Figure C After sterilization (right side), surfaces have changed with a less structured profile, larger deeps and smoother peaks.
